# P-567. Baseline Patient and Clinician Results for the Pilot Project I-CARE-4-PAH (Intervention for Collaborative Care to Assess Risk and Eliminate polyPharmacy, Falls, and Fragility Fractures for People Aging with HIV)

**DOI:** 10.1093/ofid/ofae631.765

**Published:** 2025-01-29

**Authors:** Julie A Womack, Chloe Johnson, Karina Danvers, Evelyn Hsieh, Michael Lauro, Richard Marottoli, Anne Murphy, Erika Payne, Lisa Sheehan, Wynnett Stewart, Michael D Virata, Lydia A Barakat

**Affiliations:** Yale School of Nursing and VA Connecticut Healthcare System, West Haven, Connecticut; Yale School of Medicine, New Haven, Connecticut; Yale School of Medicine, New Haven, Connecticut; Yale University School of Medicine and VA Connecticut Health System, West Haven, Connecticut; Yale School of Medicine, New Haven, Connecticut; Yale School of Medicine and VA Connecticut Healthcare System, New Haven, Connecticut; Yale New Haven Health, New Haven, Connecticut; Yale New Haven Health, New Haven, Connecticut; Yale New Haven Health, New Haven, Connecticut; Yale New Haven Health, New Haven, Connecticut; Yale University School of Medicine, New Haven, Connecticut; Yale School of Medicine, New Haven, Connecticut

## Abstract

**Background:**

More than half of people living with HIV in the United States are 50 years of age or older. Understanding how best to assess and address conditions associated with aging in HIV primary care is important for this population.

**Methods:**

We trained clinicians in an academic HIV clinic to assess and address the 4F’s (polypharmacy, Falls, and Fragility Fractures) in their patients aging with HIV (PAH). We share results of this screening as well as key knowledge, attitudes, and practices (KAP) from the pre-test assessment of our clinician champions.

**Results:**

126 patients 50+ years of age were enrolled. The mean age is 63±6 years. 49% were assigned female at birth and 1% are transwomen. 48% identify as Black, 48% White, and 14% Hispanic. 30% smoke tobacco, and 30% report ongoing marijuana use. Patients take 10±5 medications; 25% have hyperpolypharmacy ( >15 medications), and 66% are on potentially inappropriate medications (PIMs). At least a quarter of patients have peripheral neuropathy: 25% have decreased sensation on the plantar surface of either foot; 35% have decreased vibratory sense on the right great toe,37% on the left. 42% reported a fall/near-fall in the past year; 10% experienced a serious fall leading to a visit with a healthcare provider. Mean risk of a serious fall in the next year is moderate: 4.05%±4.13%. 34% reported a fracture as an adult. Mean FRAX scores indicate a low 10-year risk for fragility fracture (major osteoporotic fracture: 8.71%±6.95%; hip fracture 2.11%±3.58%).

Eleven HIV clinicians are engaged with this project. Most (73%) regularly perform medication reconciliation. 55% report asking about falls only if the patient brings it up. 18% have never assessed their patients for fracture risk, and 36% have done so once.

**Conclusion:**

Polypharmacy, peripheral neuropathy, PIMs, falls, and fractures are important intersectional risk factors for adverse outcomes in PAH and are inconsistently assessed by clinicians. Medication reconciliation is an important first step in identifying polypharmacy. Deprescribing, identifying patients with peripheral neuropathy, and discussing fall prevention are key foci for future interventions. Providing training resources for HIV clinicians to assess and prevent the 4F’s is crucial and feasible.

**Disclosures:**

**Richard Marottoli, MD, MPH**, The Hartford: Advisory Panel on Older Driver Safety **Michael D. Virata, MD, FACP**, Janssen: Advisor/Consultant|Janssen: Honoraria|ViiV Pharmaceuticals: Advisor/Consultant|ViiV Pharmaceuticals: Honoraria

